# 2-Amino-5-bromo­pyridin-1-ium (2-amino-5-bromo­pyridine-κ*N* ^1^)trichloridozincate

**DOI:** 10.1107/S1600536813011884

**Published:** 2013-05-04

**Authors:** Fitriani, Kanidtha Hansongnern, Nararak Leesakul, Chaveng Pakawatchai, Saowanit Saithong

**Affiliations:** a Department of Chemistry and Center of Excellence for Innovation in Chemistry, Faculty of Science, Prince of Songkla University, Hat Yai, Songkhla 90112, Thailand

## Abstract

The structure of the title salt, (C_5_H_6_BrN_2_)[ZnCl_3_(C_5_H_5_BrN_2_)], consists of discrete 2-amino-5-bromo­pyridin-1-ium cations and distorted tetra­hedral (2-amino-5-bromo­pyridine)­tri­chlorido­zincate anions. In the crystal, the complex anions and cations are linked *via* N—H⋯Cl hydrogen bonds into layers parallel to (101). Short Br⋯Cl contacts of 3.4239 (11) and 3.4503 (12) Å are observed, as well as π–π stacking inter­actions between the pyridine and pyridinium rings, with alternating centroid-to-centroid distances of 3.653 (2) and 3.845 (2) Å.

## Related literature

For background to the chemistry of substituted pyridines, see: Janiak *et al.* (1999 ▶); Hubrich *et al.* (2010 ▶); Wei *et al.* (2012 ▶). For the biological activities and electrochemical properties of pyridine derivatives, see: Jo *et al.* (2004 ▶); Xiao *et al.* (2012 ▶).
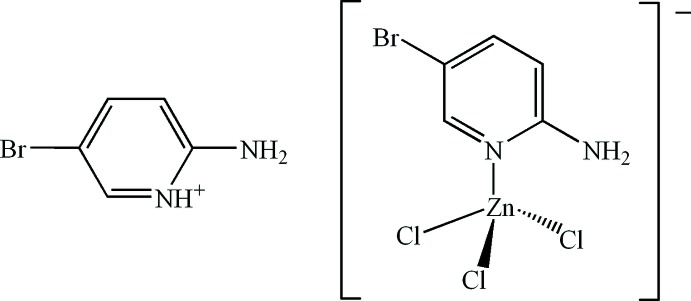


## Experimental

### 

#### Crystal data

(C_5_H_6_BrN_2_)[ZnCl_3_(C_5_H_5_BrN_2_)]
*M* *_r_* = 518.77
Monoclinic, 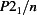

*a* = 9.4238 (4) Å
*b* = 13.6544 (6) Å
*c* = 13.5679 (6) Å
β = 104.349 (1)°
*V* = 1691.40 (13) Å^3^
*Z* = 4
Mo *K*α radiation
μ = 6.64 mm^−1^
*T* = 293 K
0.26 × 0.16 × 0.08 mm

#### Data collection

Bruker APEX CCD area-detector diffractometer
Absorption correction: multi-scan (*SADABS*; Bruker, 2003 ▶) *T*_min_ = 0.286, *T*_max_ = 0.576
18043 measured reflections
2979 independent reflections
2491 reflections with *I* > 2σ(*I*)
*R*_int_ = 0.034

#### Refinement

*R*[*F* ^2^ > 2σ(*F* ^2^)] = 0.033
*wR*(*F* ^2^) = 0.086
*S* = 1.02
2979 reflections
196 parameters
5 restraints
H atoms treated by a mixture of independent and constrained refinement
Δρ_max_ = 1.23 e Å^−3^
Δρ_min_ = −0.40 e Å^−3^

### 

Data collection: *SMART* (Bruker, 1998 ▶); cell refinement: *SAINT* (Bruker, 2003 ▶); data reduction: *SAINT*; program(s) used to solve structure: *SHELXS97* (Sheldrick, 2008 ▶); program(s) used to refine structure: *SHELXL97* (Sheldrick, 2008 ▶); molecular graphics: *Mercury* (Macrae *et al.*, 2008 ▶); software used to prepare material for publication: *SHELXTL* (Sheldrick, 2008 ▶), *PLATON* (Spek, 2009 ▶) and *publCIF* (Westrip, 2010 ▶).

## Supplementary Material

Crystal structure: contains datablock(s) I, global. DOI: 10.1107/S1600536813011884/wm2739sup1.cif

Structure factors: contains datablock(s) I. DOI: 10.1107/S1600536813011884/wm2739Isup2.hkl

Additional supplementary materials:  crystallographic information; 3D view; checkCIF report

## Figures and Tables

**Table 1:** Hydrogen-bond geometry (Å, °)

*D*—H⋯*A*	*D*—H	H⋯*A*	*D*⋯*A*	*D*—H⋯*A*
N2—H2*A*⋯Cl1	0.87 (2)	2.50 (3)	3.315 (4)	156 (4)
N2—H2*B*⋯Cl2^i^	0.87 (2)	2.43 (2)	3.288 (4)	169 (5)
N3—H3*A*⋯Cl1^ii^	0.87 (2)	2.75 (4)	3.309 (4)	124 (3)
N3—H3*A*⋯Cl1^iii^	0.87 (2)	2.74 (4)	3.297 (4)	123 (4)
N4—H4*A*⋯Cl3^iii^	0.88 (2)	2.49 (3)	3.328 (4)	159 (4)
N4—H4*B*⋯Cl2^iv^	0.87 (2)	2.46 (3)	3.295 (4)	160 (4)

Symmetry codes: (i) 
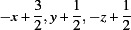
; (ii) 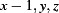; (iii) 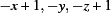; (iv) 
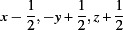
.

**Fig. 1.**
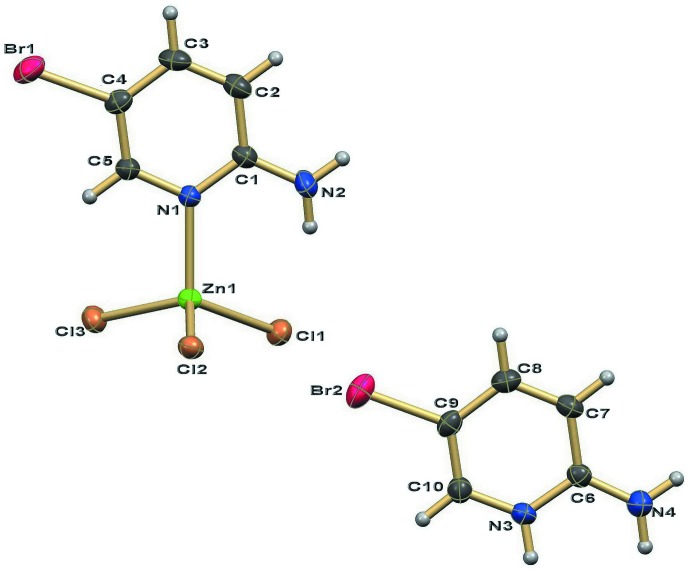
The molecular entities of cation and anion in the structure of [C5H6BrN2]+.[ZnCl3(C5H5BrN2)]-, drawn with anisotropic displacement ellipsoids at the 30% probability level.

**Fig. 2.**
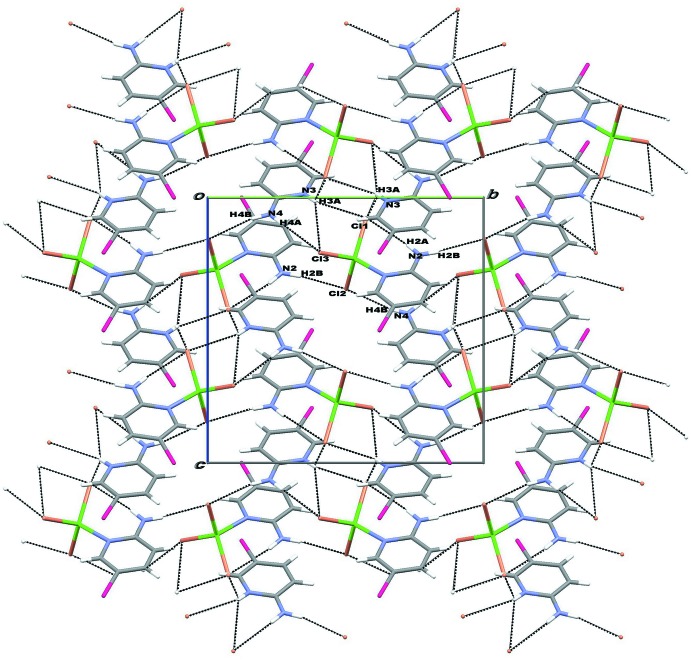
The inter- and intra-molecular hydrogen bonding interactions in [C5H6BrN2]+.[ZnCl3(C5H5BrN2)]-, as viewed down the a axis.

**Fig. 3.**
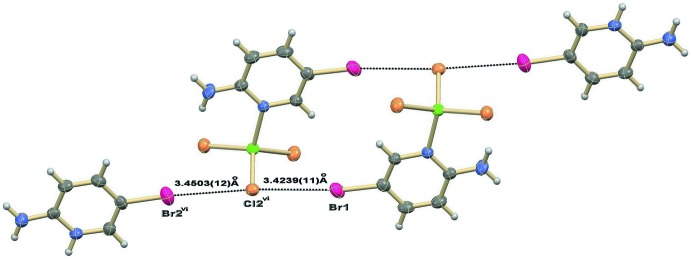
The Br···Cl short contacts in [C5H6BrN2]+.[ZnCl3(C5H5BrN2)]-, as viewed down the a axis [symmetry code: (vi) -x + 2, -y, -z].

**Fig. 4.**
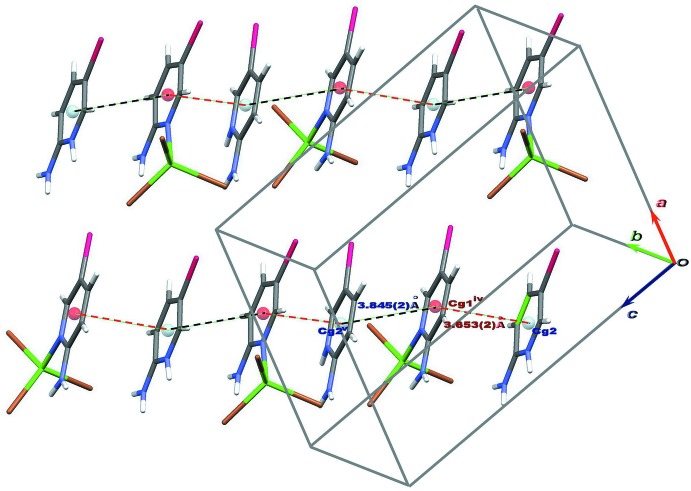
The one-dimensional inter-molecular π–π stacking in [C5H6BrN2]+.[ZnCl3(C5H5BrN2)]- [symmetry codes: (iv) x - 1/2, -y + 1/2, z + 1/2; (v) x + 1/2, -y + 1/2, z + 1/2).

## supplementary crystallographic information

### Comment

In recent years, pyridine and its derivatives were found to be suitable ligands for transition metal coordination compounds involving Fe^II^, Ni^II^ (Janiak *et al.*, 1999); Cu^II^, Co^II^ (Hubrich *et al.*, 2010) and Zn^II^ (Wei *et al.*, 2012). Several pyridine derivatives play important roles in biological activities (Jo *et al.*, 2004) or have interesting electrochemical properties (Xiao *et al.*, 2012).

In the present work, 2-amino-5-bromopyridine was reacted with ZnCl_2_ in acidic solution, forming the pyridinium cation, [C_5_H_6_BrN_2_]^+^, accompanied by the complex anion, [ZnCl_3_(C_5_H_5_BrN_2_]^-^ (Fig. 1). In the anion, the Zn^II^ atom is in a distorted tetrahedral coordination geometry, bonded to the N atom of the pyridine ring of neutral 2-amino-5-bromopyridine and to three Cl atoms. The amino group is not involved in metal coordination. The Zn1—N1 bond length is 2.052 (3) Å, and the three Zn—Cl bond lengths are Zn1—Cl1 = 2.2494 (11) Å; Zn1—Cl2 = 2.2691 (11) Å; Zn1—Cl3 = 2.2499 (11) Å. The angles around the Zn^II^ atom range from 104.99 (9) to 114.22 (4)°, indicating a slight deviation from the the ideal tetrahedral angles.

Both intra- and inter-molecular N—H···Cl hydrogen-bonding interactions are observed (Fig. 2). The only intramolecular hydrogen bond is in the complex anion, namely between the N2 atom of the amino group and the Cl1 atom [N2—H2A···Cl1, N2···Cl1 = 3.315 (4) Å]. The N2 donor is also connected to the Cl2^i^ atom (i: -*x* + 3/2, *y* + 1/2, -*z* + 1/2) of an adjacent anionic complex as an inter-molecular interaction [N2—H2B···Cl2^i^, N2···Cl2^i^ = 3.288 (4) Å]. Other inter-molecular hydrogen bonds are found between the *N*-amino group of the cation and Cl atoms of different anionic complexes [N4—H4A···Cl3^iii^, N4···Cl3^iii^ = 3.328 (4) Å; iii: -*x* + 1, -*y*, -*z* + 1; N4—H4B···Cl2^iv^, N4···Cl2^iv^ = 3.295 (4) Å; iv: *x* - 1/2, -*y* + 1/2, *z* + 1/2]. In addition, inter-molecular hydrogen bonding of the type N—H···Cl is also found among the protonated N atom of the cation and other two Cl atoms of two nearby anionic complexes [N3—H3A···Cl1^ii^, N3···Cl1^ii^ = 3.309 (4) Å; ii: *x* - 1, *y*, *z*; N3—H3A···Cl1^iii^, N3···Cl1^iii^ = 3.297 (4) Å]. All these inter-molecular hydrogen-bonding interactions generate a layer-like arrangement parallel to (101).

A short halogen···halogen contact of the type Br···Cl is found between the Br1 atom of the 2-amino-5-bromopyridine ligand of the complex anion and the Cl2 atom of an adjacent complex anion [Br1···Cl2 = 3.4239 (11) Å]. Furthermore, the Br2 atom of the neighbouring 2-amino-5-bromopyridinium cation also has a short contact to this Cl2 atom with a Br2···Cl2 distance of 3.4503 (12) Å as depicted in Fig. 3.

The crystal packing is futher stabilized by π–π stacking interactions between the pyridine ring (*Cg*1) of the anionic complex and the pyridinium ring (*Cg*2) with alternating centroid···centroid distances of *Cg*2···*Cg*1 = 3.653 (2) Å and *Cg*1···*Cg*2 = 3.845 (2) Å (Fig. 4). These interactions generate π–π stacking chains parallel to [101]. All in all, the stability of the crystal packing is governed by various interactions, including N—H···Cl hydrogen bonds, Br···Cl short contacts and π–π stacking, respectively.

### Experimental

The title compound was synthesized by dissolving zinc chloride (480 mg, 3 mmol) in methanol (10 ml) which was added to a methanolic solution of 2-amino-5-bromopyridine (519 mg, 3 mmol). The reaction mixture was stirred in the presence of a few drops of concentrated hydrochloric acid. The final pH of the solution was adjusted to 3. After stirring for 5 h, the resulting solution was filtered off and the filtrate was left for slow evaporation of the solvent at ambient temperature. After several days, brown crystals suitable for X-ray diffraction analysis were collected by filtration, washed several times with methanol and dried in a desiccator over silica gel.

The analytical data of the zinc(II) complex are given as follows. [C_5_H_6_BrN_2_]^+^.[ZnCl_3_(C_5_H_5_BrN_2_)]^-^. Yield 0.42 g, 27%. m.p. 461–463 K. Anal. Calcd. for [C_5_H_6_BrN_2_]^+^.[ZnCl_3_(C_5_H_5_BrN_2_)]^-^: C 23.15, H 2.14, N 10.80%. Found: C 23.31, H 2.09, N 10.64%.

### Refinement

All carbon H-atoms of were placed in calculated positions (C—H = 0.93 Å) and were included in the refinement in the riding-model approximation, with *U*_iso_(H) = 1.2*U*_eq_(C). The H atoms of all N atoms were located in a difference map and were restrained to N—H = 0.88 (2) Å with *U*_iso_(H) = 1.2*U*_eq_(N).

### Crystal data

**Table:**

(C_5_H_6_BrN_2_)[ZnCl_3_(C_5_H_5_BrN_2_)]	*F*(000) = 1000
*M**_r_* = 518.77	*D*_x_ = 2.037 Mg m^−^^3^
Monoclinic, *P*2_1_/*n*	Mo *K*α radiation, λ = 0.71073 Å
Hall symbol: -P 2yn	Cell parameters from 4332 reflections
*a* = 9.4238 (4) Å	θ = 2.2–24.1°
*b* = 13.6544 (6) Å	µ = 6.64 mm^−^^1^
*c* = 13.5679 (6) Å	*T* = 293 K
β = 104.349 (1)°	Block, brown
*V* = 1691.40 (13) Å^3^	0.26 × 0.16 × 0.08 mm
*Z* = 4	

### Data collection

**Table:**

Bruker APEX CCD area-detector diffractometer	2979 independent reflections
Radiation source: fine-focus sealed tube	2491 reflections with *I* > 2σ(*I*)
Graphite monochromator	*R*_int_ = 0.034
0.3° ω scans	θ_max_ = 25.0°, θ_min_ = 2.2°
Absorption correction: multi-scan (*SADABS*; Bruker, 2003)	*h* = −11→11
*T*_min_ = 0.286, *T*_max_ = 0.576	*k* = −16→16
18043 measured reflections	*l* = −16→16

### Refinement

**Table:**

Refinement on *F*^2^	Primary atom site location: structure-invariant direct methods
Least-squares matrix: full	Secondary atom site location: difference Fourier map
*R*[*F*^2^ > 2σ(*F*^2^)] = 0.033	Hydrogen site location: inferred from neighbouring sites
*wR*(*F*^2^) = 0.086	H atoms treated by a mixture of independent and constrained refinement
*S* = 1.02	*w* = 1/[σ^2^(*F*_o_^2^) + (0.0386*P*)^2^ + 2.1357*P*] where *P* = (*F*_o_^2^ + 2*F*_c_^2^)/3
2979 reflections	(Δ/σ)_max_ < 0.001
196 parameters	Δρ_max_ = 1.23 e Å^−^^3^
5 restraints	Δρ_min_ = −0.40 e Å^−^^3^

### Special details

**Table:**

Geometry. All e.s.d.'s (except the e.s.d. in the dihedral angle between two l.s. planes) are estimated using the full covariance matrix. The cell e.s.d.'s are taken into account individually in the estimation of e.s.d.'s in distances, angles and torsion angles; correlations between e.s.d.'s in cell parameters are only used when they are defined by crystal symmetry. An approximate (isotropic) treatment of cell e.s.d.'s is used for estimating e.s.d.'s involving l.s. planes.
Refinement. Refinement of *F*^2^ against ALL reflections. The weighted *R*-factor *wR* and goodness of fit *S* are based on *F*^2^, conventional *R*-factors *R* are based on *F*, with *F* set to zero for negative *F*^2^. The threshold expression of *F*^2^ > σ(*F*^2^) is used only for calculating *R*-factors(gt) *etc*. and is not relevant to the choice of reflections for refinement. *R*-factors based on *F*^2^ are statistically about twice as large as those based on *F*, and *R*-factors based on ALL data will be even larger.

### Fractional atomic coordinates and isotropic or equivalent isotropic displacement parameters (Å^2^)

**Table:**

	*x*	*y*	*z*	*U*_iso_*/*U*_eq_	
Zn1	0.84827 (5)	0.03227 (3)	0.27004 (3)	0.04549 (14)	
N1	0.9443 (3)	0.1421 (2)	0.2061 (2)	0.0424 (7)	
N2	0.8519 (5)	0.2686 (3)	0.2841 (3)	0.0656 (10)	
H2A	0.816 (5)	0.226 (3)	0.320 (3)	0.079*	
H2B	0.843 (5)	0.3311 (17)	0.295 (4)	0.079*	
N3	0.1587 (4)	0.1505 (2)	0.4873 (3)	0.0519 (8)	
H3A	0.104 (4)	0.112 (3)	0.513 (3)	0.062*	
N4	0.0611 (5)	0.2750 (3)	0.5635 (3)	0.0642 (10)	
H4A	0.019 (5)	0.234 (3)	0.597 (3)	0.077*	
H4B	0.056 (5)	0.3368 (17)	0.579 (4)	0.077*	
Cl1	0.81449 (12)	0.07487 (8)	0.42275 (8)	0.0559 (3)	
Cl2	0.63220 (11)	0.00231 (7)	0.15590 (7)	0.0509 (2)	
Cl3	1.00795 (13)	−0.09311 (7)	0.29181 (8)	0.0589 (3)	
C1	0.9311 (4)	0.2387 (3)	0.2208 (3)	0.0461 (9)	
C2	1.0012 (5)	0.3065 (3)	0.1699 (3)	0.0588 (11)	
H2	0.9933	0.3733	0.1810	0.071*	
C3	1.0794 (5)	0.2747 (3)	0.1055 (3)	0.0614 (12)	
H3	1.1252	0.3191	0.0716	0.074*	
C4	1.0908 (4)	0.1746 (3)	0.0904 (3)	0.0521 (10)	
C5	1.0238 (4)	0.1116 (3)	0.1420 (3)	0.0461 (9)	
H5	1.0333	0.0446	0.1326	0.055*	
C6	0.1411 (4)	0.2466 (3)	0.5014 (3)	0.0469 (9)	
C7	0.2104 (5)	0.3113 (3)	0.4476 (3)	0.0538 (10)	
H7	0.2005	0.3786	0.4547	0.065*	
C8	0.2913 (4)	0.2767 (3)	0.3856 (3)	0.0552 (10)	
H8	0.3366	0.3199	0.3501	0.066*	
C9	0.3068 (4)	0.1754 (3)	0.3750 (3)	0.0528 (10)	
C10	0.2379 (5)	0.1142 (3)	0.4251 (3)	0.0536 (10)	
H10	0.2447	0.0469	0.4171	0.064*	
Br1	1.19546 (6)	0.12257 (4)	0.00001 (4)	0.07623 (18)	
Br2	0.42174 (6)	0.12277 (5)	0.29263 (4)	0.07970 (19)	

### Atomic displacement parameters (Å^2^)

**Table:**

	*U*^11^	*U*^22^	*U*^33^	*U*^12^	*U*^13^	*U*^23^
Zn1	0.0510 (3)	0.0391 (2)	0.0496 (3)	−0.00250 (19)	0.0187 (2)	0.00095 (19)
N1	0.0439 (17)	0.0370 (16)	0.0489 (18)	−0.0010 (13)	0.0163 (14)	0.0004 (13)
N2	0.086 (3)	0.040 (2)	0.079 (3)	0.0100 (19)	0.036 (2)	−0.0003 (19)
N3	0.056 (2)	0.0417 (19)	0.061 (2)	−0.0096 (15)	0.0204 (17)	−0.0032 (16)
N4	0.076 (3)	0.055 (2)	0.070 (2)	−0.005 (2)	0.033 (2)	−0.006 (2)
Cl1	0.0633 (6)	0.0595 (6)	0.0492 (6)	0.0011 (5)	0.0218 (5)	−0.0012 (5)
Cl2	0.0517 (6)	0.0443 (5)	0.0568 (6)	−0.0039 (4)	0.0137 (5)	0.0020 (4)
Cl3	0.0694 (7)	0.0455 (5)	0.0646 (6)	0.0112 (5)	0.0219 (5)	0.0063 (5)
C1	0.047 (2)	0.039 (2)	0.049 (2)	−0.0024 (16)	0.0062 (18)	0.0020 (17)
C2	0.072 (3)	0.042 (2)	0.060 (3)	−0.008 (2)	0.011 (2)	0.0033 (19)
C3	0.070 (3)	0.054 (3)	0.058 (3)	−0.019 (2)	0.012 (2)	0.011 (2)
C4	0.048 (2)	0.065 (3)	0.044 (2)	−0.0064 (19)	0.0119 (18)	0.0017 (19)
C5	0.047 (2)	0.043 (2)	0.049 (2)	−0.0042 (17)	0.0143 (18)	0.0007 (17)
C6	0.043 (2)	0.045 (2)	0.050 (2)	−0.0043 (17)	0.0064 (18)	−0.0033 (17)
C7	0.058 (3)	0.040 (2)	0.062 (3)	−0.0053 (18)	0.010 (2)	0.0050 (19)
C8	0.051 (2)	0.061 (3)	0.055 (2)	−0.008 (2)	0.014 (2)	0.008 (2)
C9	0.039 (2)	0.068 (3)	0.052 (2)	0.0020 (19)	0.0130 (18)	−0.001 (2)
C10	0.052 (2)	0.052 (2)	0.057 (2)	0.0014 (19)	0.015 (2)	−0.005 (2)
Br1	0.0751 (3)	0.1016 (4)	0.0625 (3)	−0.0195 (3)	0.0369 (3)	−0.0081 (3)
Br2	0.0638 (3)	0.1060 (4)	0.0769 (4)	0.0159 (3)	0.0318 (3)	0.0004 (3)

### Geometric parameters (Å, º)

**Table:**

Zn1—N1	2.052 (3)	C2—C3	1.347 (6)
Zn1—Cl1	2.2494 (11)	C2—H2	0.9300
Zn1—Cl3	2.2499 (11)	C3—C4	1.390 (6)
Zn1—Cl2	2.2691 (11)	C3—H3	0.9300
N1—C1	1.344 (5)	C4—C5	1.359 (5)
N1—C5	1.347 (5)	C4—Br1	1.892 (4)
N2—C1	1.334 (5)	C5—H5	0.9300
N2—H2A	0.874 (19)	C6—C7	1.406 (6)
N2—H2B	0.873 (19)	C7—C8	1.354 (6)
N3—C6	1.342 (5)	C7—H7	0.9300
N3—C10	1.351 (5)	C8—C9	1.401 (6)
N3—H3A	0.868 (19)	C8—H8	0.9300
N4—C6	1.320 (6)	C9—C10	1.342 (6)
N4—H4A	0.881 (19)	C9—Br2	1.882 (4)
N4—H4B	0.874 (19)	C10—H10	0.9300
C1—C2	1.413 (6)		
			
N1—Zn1—Cl1	112.23 (9)	C2—C3—C4	119.2 (4)
N1—Zn1—Cl3	105.12 (9)	C2—C3—H3	120.4
Cl1—Zn1—Cl3	108.58 (4)	C4—C3—H3	120.4
N1—Zn1—Cl2	104.99 (9)	C5—C4—C3	118.9 (4)
Cl1—Zn1—Cl2	111.55 (4)	C5—C4—Br1	118.6 (3)
Cl3—Zn1—Cl2	114.22 (4)	C3—C4—Br1	122.5 (3)
C1—N1—C5	119.1 (3)	N1—C5—C4	122.7 (4)
C1—N1—Zn1	126.0 (3)	N1—C5—H5	118.7
C5—N1—Zn1	114.9 (2)	C4—C5—H5	118.7
C1—N2—H2A	121 (3)	N4—C6—N3	119.3 (4)
C1—N2—H2B	120 (3)	N4—C6—C7	123.9 (4)
H2A—N2—H2B	119 (5)	N3—C6—C7	116.8 (4)
C6—N3—C10	123.7 (4)	C8—C7—C6	120.6 (4)
C6—N3—H3A	116 (3)	C8—C7—H7	119.7
C10—N3—H3A	120 (3)	C6—C7—H7	119.7
C6—N4—H4A	123 (3)	C7—C8—C9	119.8 (4)
C6—N4—H4B	121 (3)	C7—C8—H8	120.1
H4A—N4—H4B	115 (5)	C9—C8—H8	120.1
N2—C1—N1	118.9 (4)	C10—C9—C8	119.2 (4)
N2—C1—C2	121.2 (4)	C10—C9—Br2	119.0 (3)
N1—C1—C2	120.0 (4)	C8—C9—Br2	121.9 (3)
C3—C2—C1	120.1 (4)	C9—C10—N3	119.9 (4)
C3—C2—H2	119.9	C9—C10—H10	120.0
C1—C2—H2	119.9	N3—C10—H10	120.0
			
Cl1—Zn1—N1—C1	−29.5 (3)	C1—N1—C5—C4	−0.6 (6)
Cl3—Zn1—N1—C1	−147.3 (3)	Zn1—N1—C5—C4	177.7 (3)
Cl2—Zn1—N1—C1	91.9 (3)	C3—C4—C5—N1	1.2 (6)
Cl1—Zn1—N1—C5	152.4 (2)	Br1—C4—C5—N1	−178.5 (3)
Cl3—Zn1—N1—C5	34.5 (3)	C10—N3—C6—N4	179.7 (4)
Cl2—Zn1—N1—C5	−86.3 (3)	C10—N3—C6—C7	0.0 (6)
C5—N1—C1—N2	179.8 (4)	N4—C6—C7—C8	179.7 (4)
Zn1—N1—C1—N2	1.7 (5)	N3—C6—C7—C8	−0.5 (6)
C5—N1—C1—C2	−0.5 (5)	C6—C7—C8—C9	−0.2 (6)
Zn1—N1—C1—C2	−178.6 (3)	C7—C8—C9—C10	1.5 (6)
N2—C1—C2—C3	−179.3 (4)	C7—C8—C9—Br2	−178.4 (3)
N1—C1—C2—C3	1.0 (6)	C8—C9—C10—N3	−2.0 (6)
C1—C2—C3—C4	−0.4 (7)	Br2—C9—C10—N3	177.8 (3)
C2—C3—C4—C5	−0.7 (6)	C6—N3—C10—C9	1.3 (6)
C2—C3—C4—Br1	179.0 (3)		

### Hydrogen-bond geometry (Å, º)

**Table:**

*D*—H···*A*	*D*—H	H···*A*	*D*···*A*	*D*—H···*A*
N2—H2*A*···Cl1	0.87 (2)	2.50 (3)	3.315 (4)	156 (4)
N2—H2*B*···Cl2^i^	0.87 (2)	2.43 (2)	3.288 (4)	169 (5)
N3—H3*A*···Cl1^ii^	0.87 (2)	2.75 (4)	3.309 (4)	124 (3)
N3—H3*A*···Cl1^iii^	0.87 (2)	2.74 (4)	3.297 (4)	123 (4)
N4—H4*A*···Cl3^iii^	0.88 (2)	2.49 (3)	3.328 (4)	159 (4)
N4—H4*B*···Cl2^iv^	0.87 (2)	2.46 (3)	3.295 (4)	160 (4)

Symmetry codes: (i) −*x*+3/2, *y*+1/2, −*z*+1/2; (ii) *x*−1, *y*, *z*; (iii) −*x*+1, −*y*, −*z*+1; (iv) *x*−1/2, −*y*+1/2, *z*+1/2.

## References

[bb1] Bruker (1998). *SMART* Bruker AXS Inc., Madison, Wisconsin, USA.

[bb2] Bruker (2003). *SAINT* and *SADABS* Bruker AXS Inc., Madison, Wisconsin, USA.

[bb3] Hubrich, M., Peukert, M., Seichter, W. & Weber, E. (2010). *Polyhedron*, **29**, 1854–1862.

[bb4] Janiak, C., Deblon, S., Wu, H., Kolm, M. J., Klüfers, P., Piotrowski, H. & Mayer, P. (1999). *Eur. J. Inorg. Chem.* pp. 1507–1521.

[bb5] Jo, Y. W., Im, W. B., Rhee, J. K., Shim, M. J., Kim, W. B. & Choi, E. C. (2004). *Bioorg. Med. Chem.* pp. 5909–5915. 10.1016/j.bmc.2004.08.025 15498667

[bb6] Macrae, C. F., Bruno, I. J., Chisholm, J. A., Edgington, P. R., McCabe, P., Pidcock, E., Rodriguez-Monge, L., Taylor, R., van de Streek, J. & Wood, P. A. (2008). *J. Appl. Cryst.* **41**, 466–470.

[bb7] Sheldrick, G. M. (2008). *Acta Cryst.* A**64**, 112–122. 10.1107/S0108767307043930 18156677

[bb8] Spek, A. L. (2009). *Acta Cryst.* D**65**, 148–155. 10.1107/S090744490804362X PMC2631630 19171970

[bb9] Wei, Z., Xie, X., Zhao, J., Huang, L. & Liu, X. (2012). *Inorg. Chim. Acta*, **387**, 277–282.

[bb10] Westrip, S. P. (2010). *J. Appl. Cryst.* **43**, 920–925.

[bb11] Xiao, X. W., He, Y. J., Sun, L. W., Wang, G. N., Shen, L. J., Fang, J. H. & Yang, J. P. (2012). *Transition Met. Chem.* pp. 1–5.

